# Bioadhesive Tannic-Acid-Functionalized Zein Coating Achieves Engineered Colonic Delivery of IBD Therapeutics via Reservoir Microdevices

**DOI:** 10.3390/pharmaceutics14112536

**Published:** 2022-11-21

**Authors:** Khorshid Kamguyan, Rolf Bech Kjeldsen, Saeed Zajforoushan Moghaddam, Melanie Randahl Nielsen, Esben Thormann, Kinga Zór, Line Hagner Nielsen, Anja Boisen

**Affiliations:** 1 Department of Health Technology, Technical University of Denmark, Ørsteds Pl., 2800 Kongens Lyngby, Denmark; 2 Department of Chemistry, Technical University of Denmark, Kemitorvet, 2800 Kongens Lyngby, Denmark

**Keywords:** 5-aminosalicylic acid, prolonged delivery, mucoadhesion, microcontainers, X-ray imaging, plant phenolics, controlled drug release

## Abstract

The biggest challenge in oral delivery of anti-inflammatory drugs such as 5-aminosalicylic acid (5-ASA) is to (i) prevent rapid absorption in the small intestine and (ii) achieve localized release at the site of inflammation in the lower gut, i.e., the colon. Here, we present an advanced biopolymeric coating comprising of tannic-acid-functionalized zein protein to provide a sustained, colon-targeted release profile for 5-ASA and enhance the mucoadhesion of the dosage form via a mussel-inspired mechanism. To enable localized delivery and provide high local concentration, 5-ASA is loaded into the microfabricated drug carriers (microcontainers) and sealed with the developed coating. The functionality and drug release profile of the coating are characterized and optimized in vitro, showing great tunability, scalability, and stability toward proteases. Further, ex vivo experiments demonstrate that the tannic acid functionalization can significantly enhance the mucoadhesion of the coating, which is followed up by in vivo investigations on the intestinal retention, and pharmacokinetic evaluation of the 5-ASA delivery system. Results indicate that the developed coating can provide prolonged colonic delivery of 5-ASA. Therefore, the here-developed biodegradable coating can be an eco-friendly substitute to the state-of-the-art commercial counterparts for targeted delivery of 5-ASA and other small molecule drugs.

## 1. Introduction

Inflammatory bowel diseases (IBD) including Crohn’s disease and ulcerative colitis are chronic illnesses affiliated with the lower part of the gastrointestinal tract (GIT) [1]. The first course of treatment is the administration of anti-inflammatory drugs such as 5-aminosalicylic acid (5-ASA, also known as mesalamine) [2,3]. For an effective course of therapy, 5-ASA must be delivered in high concentration locally to the site of the inflammation in the lower GIT, specifically the colon. However, the inability of the drug carrier to prevent premature release in the upper GIT can cause rapid absorption of 5-ASA into the blood circulation, resulting in numerous side effects and rendering the treatment ineffective [4,5,6]. In this regard, research on the next generation of drug carriers, i.e., ingestible microfabricated devices, can offer new solutions to conventional challenges in controlled colonic delivery of small molecule drugs such as 5-ASA [7].

Polymeric microcontainers (MCs) have been broadly investigated for their potential in the engineered delivery of active compounds [8]. These reservoir-based devices are loaded with an active ingredient and sealed with a polymeric lid coating [9]. The latter enables surface functionalization of the device [10], tunability of the drug release profile [11,12], and targeted delivery. In a recent study, we showed that a crosslinked chitosan coating allows MCs to deliver live probiotic bacteria locally to the colon ensuring a site-specific release profile [13]. However, such hydrophilic coatings cannot control the release of small molecule drugs due to their high water permeability and swelling in the upper GIT, which has also been manifested in the case of polysaccharide-based particulate systems [14,15,16]. Therefore, to effectively control the release of small molecule drugs such as 5-ASA, the lid coating must be developed from a hydrophobic polymer with low swelling capacity. Such properties can be found in many commercial coatings such as Eudragit^®^ and Kollicoat^®^ [17,18]. However, the use of synthetic and non-biodegradable polymers is unfavorable as they pollute the environment after leaving the body, which has led to rising research on more eco-friendly material technologies [19,20,21]. The search for a sustainable material with the aforementioned characteristics has led us to the hydrophobic zein protein [22].

Zein, a maize-derived storage protein, has been investigated for the synthesis of particulate controlled-release drug delivery systems due to its solubility in aqueous ethanol solutions and insolubility in water [23,24]. The latter allows zein-based drug delivery systems to provide time-dependent and tunable drug release profiles. Liu et al. studied zein/pectin beads for colonic drug delivery, showing a delayed release of indomethacin in vitro in the presence of pectinate enzymes [25]. In another study, Wang et al. investigated in situ polymerization of dopamine in indomethacin-encapsulating zein matrixes, which showed a sustained release of the drug under in vitro conditions [26]. More recently, Bisharat et al. showed similar results for acetylated high amylose starch/zein film coatings for colonic delivery of paracetamol tablets [27]. It was shown that the zein-based coatings were resistant to pH variations as well as the protease enzymes (pepsin and pancreatin) in the upper GIT, resulting in a prolonged release of the encapsulated drug.

An effective IBD treatment with 5-ASA, aside from a tunable sustained release profile [28,29], requires a tunable intestinal retention time of the drug carrier to have a significant effect under in vivo conditions. In this respect, it has been shown that the functionalization of zein with plant phenolics can enhance the adhesion of the polymer to a wet substrate [30,31]. Plant phenolics, such as tannic acid, can form physical/chemical crosslinks with the amino acid residues in zein while enabling a mussel-inspired adhesion to the intestinal mucosa. To the best of our knowledge, the combination of zein and plant phenolics has not been studied for increasing the mucoadhesion of a drug carrier; however, mussel-inspired adhesion has been investigated previously via catechol-functionalization of chitosan-based drug delivery systems [32,33].

**Figure 1:**
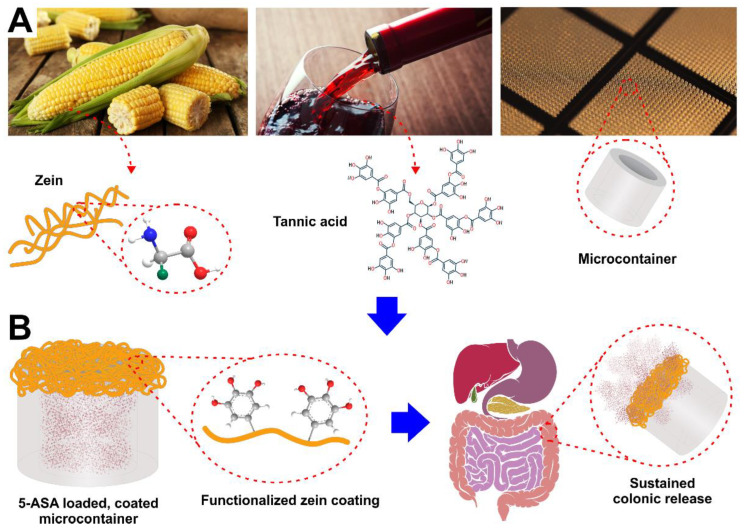
Illustrative concept of the study, which aims to combine the functionalities of zein protein, tannic acid, and MCs (**A**), creating a drug carrier for sustained colonic delivery of 5-ASA (**B**).

In this study, we developed tannic-acid-functionalized zein coatings to achieve a prolonged colonic release of 5-ASA loaded into MCs, based on the hydrophobic nature of zein and tannic-acid-mediated mucoadhesion (Figure 1A,B). Zein/tannic acid coatings were characterized and optimized in vitro, systematically evaluating the effect of pH and digestive enzymes on the release of 5-ASA from coated MCs, as well as gelatin capsules. Furthermore, mucoadhesion of the biopolymeric coating was assessed ex vivo, by tracking the zein/tannic-acid-coated MCs in the GIT of rats, to identify whether or not the developed coating can sustain the retention of the drug carriers in vivo. Finally, the release and absorption of 5-ASA from zein/tannic-acid-coated MCs were studied in vivo and compared with commercially available dosage forms of the anti-inflammatory drug.

## 2. Materials and Methods

### 2.1. Materials

5-aminosalicylic acid, zein, tannic acid, carboxymethylcellulose sodium salt (viscosity = 100 cP, 4% at 25 °C, degree of substitution = 0.81), HEPES, phosphate buffer saline (PBS), ethanol absolute, hydrochloric acid, sodium hydroxide, formic acid (98–100%), pepsin (from porcine gastric mucosa), and trypsin (from porcine pancreas) were purchased from Sigma Aldrich (St. Louis, MO, USA). Eudragit^®^ FS-100 (EFS100) was supplied by Evonik (Essen, Germany). Sylgard^®^ 184 polydimethylsiloxane (PDMS) was purchased from Dow Corning (Midland, MI, USA). SU-8 2035, SU-8 2075, and SU-8 developer were purchased from micro resist technology GmbH (Berlin, Germany). Barium Sulfide and acetonitrile (Super-gradient) were supplied by VWR International (Radnor, PA, USA). Fasted-state simulated gastric, intestinal, and colonic fluids (FaSSIF/FeSSIF/FaSSGF and FaSSCoF; detailed description is available in supporting information Section S4, provided by the manufacturer) were purchased from Biorelevant.com Ltd. (London, U.K.). Gelatin capsules (size 9) were purchased from Torpac^®^ (Fairfield, NJ, USA) and silicon (Si) wafers (ø100 mm single-side (thickness = 525 µm) polished) were obtained from Topsil Global Wafers A/S (Frederikssund, Denmark). Ultrapure water (Millipore Corporation, Billerica, MA) was used in the preparation of all solutions.

### 2.2. Preparation of Zein and Zein/Tannic Acid Solutions

Zein solutions were prepared in 90% ethanol and stirred for 2 h at room temperature. To prepare zein/tannic acid solutions, zein and tannic acid (1% *w*/*w* on zein) were added to 90% ethanol and stirred for 2 h. The pH of the solution was adjusted to 7 using NaOH 1 M to induce oxidation and thus in situ polymerization of tannic acid [30,34]. All solutions were freshly prepared for each experiment and kept in storage for a maximum of 24 h.

### 2.3. ATR-FTIR Spectroscopy

Fourier-transform infrared (FTIR) spectra of zein and zein/tannic acid films were collected using a Spectrum 100 FT-IR spectrometer (PerkinElmer, Waltham, MA, USA) equipped with an attenuated total reflectance (ATR) accessory in the wavenumber range from 4000 to 650 cm^−^^1^ with a resolution of 4 cm^−^^1^ and 25 scans. Free-standing films were prepared by solvent casting 5% *w*/*v* zein and zein/tannic acid solutions (containing 10% glycerol relative to the zein content) into Petri dishes and were dried at room temperature overnight.

### 2.4. Ellipsometry

The dry and wet thicknesses of films were determined by spectroscopic ellipsometry (M-2000 U 245–1000 nm, J.A. Woollam Co., Inc., Lincoln, NE, USA). For this, 0.5% *w*/*v* zein and zein/tannic acid solutions were spin-coated onto silica wafers for 60 s using 4000 rpm speed. This optimized procedure allowed for obtaining films with a relatively small surface roughness/heterogeneity suitable for ellipsometry studies. Two sets of ellipsometric experiments were conducted to investigate the pH stability and swelling properties of the films. To assess the pH stability, the dry thickness of the films was measured before and after immersion (1 h, 250 rpm stirring) in aqueous solutions that mimic the pH of the gastrointestinal fluids (50 mM NaCl, pH adjusted to 1.6, 6.5, or 7.8 using HCl and NaOH). The instrument’s standard liquid cell was used for measurements in solution to investigate the swelling properties of the coatings at various pH conditions. The liquid cell was sequentially loaded with aqueous solutions of pH 1.6, 6.5, 7.8, and 1.6, giving 15 min of equilibration time for each step. The refractive index of the coatings was modeled using a Cauchy relation for transparent films (n(λ) = A + Bλ^−2^). All experiments were performed in triplicate at room temperature.

### 2.5. Fabrication of MCs

MCs were manufactured from SU-8 negative photoresist via a UV-lithography-based fabrication scheme as previously reported [13,35]. SU-8 2035 and SU-8 2075 were spin-coated on Si wafers and subjected to UV exposure using a Karl Süss Mask Aligner MA6 (Süss MicroTec, Garching, Germany). Subsequently, the substrates were developed in two mr-Dev 600 baths followed by flushing and drying. The Si substrates holding the fabricated MCs were then cut into 1.2 × 1.2 cm chips using an automatic dicing saw (DAD 321, DISCO, Tokyo, Japan). To facilitate the detachment of MCs from the Si substrates (for in vivo studies), the Si wafers were coated with a release layer of 5 nm Ti and 20 nm Au.

### 2.6. Drug Loading and Deposition of Coatings

5-ASA was loaded into MCs by a previously established technique using PDMS to mask the areas in between the containers [35]. In short, PDMS resin and crosslinker were mixed at a ratio of 10:1, poured on the Si chips holding the MCs, and crosslinked at 70 °C for 30 min. The masked containers were then loaded using a spatula to press the drug powder on top of the chip, then the PDMS mask was gently peeled off the substrate using a tweezer.

5-ASA-loaded MCs, as well as gelatin capsules, were sealed with polymeric coatings using an ultrasonic spray coater (Sono-Tek, Milton, NY, USA) with an AccuMist™ nozzle. Zein and zein/tannic acid solutions (5% *w*/*v* zein) in ethanol 90%, as well as EFS100 solutions in ethanol 90% (1% *w*/*v*), were spray coated at 40 °C with an atomizer pressure of 0.02 kPa, nozzle speed of 20 mm·s^−^^1^, 0.07 mL/min infuse rate, and generator power of 1.3 W.

To obtain different thicknesses of the zein coating, 5-ASA-loaded MCs were spray coated for 10, 20, 60, 100, 140, and 180 loops to obtain thicknesses of 10 µm (Z-10), 20 µm (Z-20), 80 µm (Z-80), 120 µm (Z-120), 170 µm (Z-170), and 220 µm (Z-220), respectively. Subsequently, loaded MCs as well as capsules were spray coated with zein/tannic acid for 60 loops to yield an 80 µm thick coating (ZT-80). EFS100 was spray coated for 180 loops to yield a 70 µm thick coating. The coated MCs and capsules were kept at room temperature to dry overnight.

Characterization of MCs during the loading/coating stage was performed using scanning electron microscopy with a TM3030Plus tabletop microscope (Hitachi High Technologies Europe GmbH, Krefeld, Germany) with a voltage of 15 keV and bright field microscopy using a Nikon Eclipse L200 (Nikon Metrology, Tokyo, Japan).

### 2.7. In Vitro Drug Release Measurements

Release of 5-ASA from MCs and gelatin capsules was monitored using a µ-Diss profiler (pION INC, Woburn, MA, USA). To obtain release profiles in simulated gastrointestinal conditions, aqueous release media were prepared as follows: simulated gastric medium (SGM) was prepared by adding 0.06 g/L of FaSSIF/FeSSIF/FaSSGF to 25 mM HCl in ultrapure water. The pH of SGM was adjusted to 1.6 using 1 M HCl. Simulated small intestinal medium (SIM) was prepared by adding 2.24 g/L of FaSSIF/FeSSIF/FaSSGF to 50 mM HEPES buffer in ultrapure water and the pH of the solution was adjusted to 6.5 using 1 M HCl and 1 M NaOH. Simulated colonic medium (SCM) was obtained by adding 0.34 g/L of FaSSCoF to 50 mM HEPES buffer in ultrapure water and the pH was adjusted to 7.8.

The media containing GIT enzymes was prepared by adding 0.5 mg/mL of pepsin to the SGM and 0.1 mg/mL of trypsin to the SIM. To prepare SCM containing colonic bacterial enzymes, feces were collected from Sprague–Dawley rats and suspended in the prepared medium. The suspension was then centrifuged at 500 rpm for 15 min, after which the supernatants were re-centrifuged at 14,000 rpm for 30 min. The supernatants were used as the simulated colonic fluid.

Calibration curves were obtained at 330 nm for SGM and at 370 nm for SIM and SCM before each experiment. Chips holding loaded and coated MCs as well as gelatin capsules were attached to magnetic stirrers and cumulative release of the loaded 5-ASA was monitored in 10 mL of the biorelevant media in sequential steps (1 h in SGM, 3 h in SIM, and 20 h in SCM). UV-visible spectra of the released drug were monitored in real time using UV probes with a path length of 5 mm. All experiments were performed in triplicate at 37 °C with a stirring rate of 100 rpm.

### 2.8. Ex Vivo Mucoadhesion Assay

Porcine intestinal tissue was obtained from 15–16-week-old Landrace × Yorkshire × Duroc (LYD) pigs under the license number DK-10-13-oth-736416. Segments of the jejunum of pigs were dissected along the intestinal axis and cut into 50 × 50 mm pieces. The tissue pieces were washed with PBS to remove the intestinal contents and non-digested food from the lumen, as well as the transient and non-adherent layer of mucus. The underlying connective tissue was then removed to isolate the mucosal membrane, which was mounted onto a holder stage and immersed in a container with 200 mL of ultrapure water.

Adhesion of polymers to porcine mucosa was studied using a TA.XTplus texture analyzer (Stable Micro Systems Ltd., Godalming, U.K.). Zein and zein/tannic acid solutions in ethanol 90% (5% *w*/*v*), CMC in ultrapure water (1% *w*/*v*), and EFS100 in ethanol 90% (1% *w*/*v*) were spin-coated on 10 × 10 mm Si substrates to obtain a 15 μm thick coating. The coated Si substrates were then attached to a cylindrical probe with a 10 mm diameter. Experiments were performed using a descending speed of 1 mm·s^−1^, a contact time of 5 min, an applied force of 100 g, and an ascending speed of 0.05 mm·s^−1^.

Two parameters were used to characterize the mucoadhesion of the polymeric films: detachment force/surface area, which was calculated using the peak force of the force–displacement curves; and the work of adhesion, which was calculated based on the area under the force–displacement curves. All experiments were carried out at room temperature with four replicates.

### 2.9. In Vivo Evaluation of Intestinal Retention

All animal experiments were performed according to the Danish and European guidelines for animal housing and care and the euthanasia procedures followed the humane endpoints stated in welfare norms at the National Food Institute at the Technical University of Denmark. The experiments were conducted under the approval of the local institutional Animal Welfare Committee (license number: 2020-15-0201-00610) in compliance with the Danish laws regulating experiments on animals and EC Directive 2010/63/EU.

A total of 30 male Sprague–Dawley rats (with a weight of 284.2 ± 11.3 g) were housed with three animals in each cage and had access to water and standard feed ad libitum. Animals were divided into two groups dosed with gelatin capsules (one capsule/rat) containing either ZT-80- or EFS100-coated MCs loaded with BaSO4 (as a contrast agent) topped with a thin layer of 5-ASA. The animals were sacrificed at 3 h, 10 h, 14 h, 18 h, and 24 h after dosage.

### 2.10. CT Scanning and Planar X-ray Imaging

The sacrificed rats were CT scanned using a Nikon XT H 225 (Nikon Metrology, Tokyo, Japan) to obtain 3D visualization of the location of MCs, which was obtained from single planar scans using 1572 projections with 2 frames per projection and an exposure time of 1 s. X-rays were produced using a power of 30 W, a voltage of 70 kV (current of 0.43 mA), and a voxel size of 231.566 µm. A Feldkamp, Davis, and Kress algorithm [36] was used for the following reconstruction in the software provided with the CT scanner system (CT Pro 3D, Nikon Metrology, Tokyo, Japan). Lastly, a 3D visualization and analysis software (Avizo, Thermo Fisher Scientific Inc., Waltham, MA, USA) was used to process the data.

Subsequently, the sacrificed rats were dissected, and their GIT was removed to investigate the exact location of the MCs inside the body as well as to quantify the number of MCs at specific sections of the GIT by planar X-ray imaging. X-rays were generated using a power of 30 W and a voltage of 70 kV (current of 0.43 mA) and the distance between the x-ray probe and the samples was set to obtain a magnification of 2. Planar X-ray images, including a background signal for shading correction, were acquired using 8 frames and an exposure time of 1 s. An image processing software (ImageJ, freeware) was used for the shading corrections and for the subsequent manual counting of the MCs throughout the entire GIT.

### 2.11. In Vivo Evaluation of 5-ASA Delivery

Animal experiments for oral delivery of 5-ASA were performed according to the regulations described in the previous section. A total of 18 male Sprague–Dawley rats (with a weight of 348.5 ± 13.3 g) were housed in individual cages with access to water and standard feed ad libitum. The rats were divided into three groups and dosed with an average amount of 18.9 ± 2.9 mg/kg of the drug in the form of either free 5-ASA powder, ZT-MCs, or Pentasa^®^ loaded into gelatin capsules using a polyurethane feeding tube (Instech Laboratories Inc., Plymouth Meeting, PA, USA). Blood samples were taken from the lateral tail vein before dosing and at 1 h, 3 h, 5 h, 7 h, 9 h, 25 h, and 30 h after dosage. The rats were then euthanized by CO2 gas and decapitation. Blood samples were centrifuged at 14,000× *g* for 10 min immediately after collection to obtain plasma, which was stored at −20 °C until analysis.

### 2.12. Quantitative Analysis of 5-ASA in Plasma Samples by High-Performance Liquid Chromatography–Mass Spectrometry (HPLC-MS)

Plasma samples were analyzed using Agilent 1260 Infinity II HPLC system fitted with a G6465 Ultivo Triple Quadrupole and a 1260 Infinity II Diode Array Detector (Agilent Technologies, Santa Clara, CA, USA). Analysis was performed using a Kinetex 5 µ C18, 100A 150 × 4.6 µm column (Phenomenex ApS, Nordic Region, Værløse, Denmark) at a flow rate of 0.2 mL/min, a column oven temperature of 400 °C, an autosampler temperature of 180 °C, and an inject volume of 5 µL. A gradient mobile phase program was used for the analysis using purified water +0.1% formic acid (solvent A) and acetonitrile +0.1% formic acid (solvent B) as follows: 0–5.5 min, 100% solvent A; 5.5–7 min, 95% solvent B; 7–12 min, 100% solvent A. Sample preparation was performed by addition of 25 µL of plasma to 175 µL of acetonitrile, which was then centrifuged at 14,000× *g* for 10 min. The supernatants were added to HPLC vials for analysis.

### 2.13. Data Analysis

Data are presented as mean ± standard deviation or standard error. Statistical analysis was conducted using OriginLab software version 2018b (OriginLab Corporation, Northampton, MA, USA). An unpaired t-test was used to calculate *p*-values, where *p* ≤ 0.05 was considered statistically significant.

## 3. Results

### 3.1. Characterization

**Figure 2:**
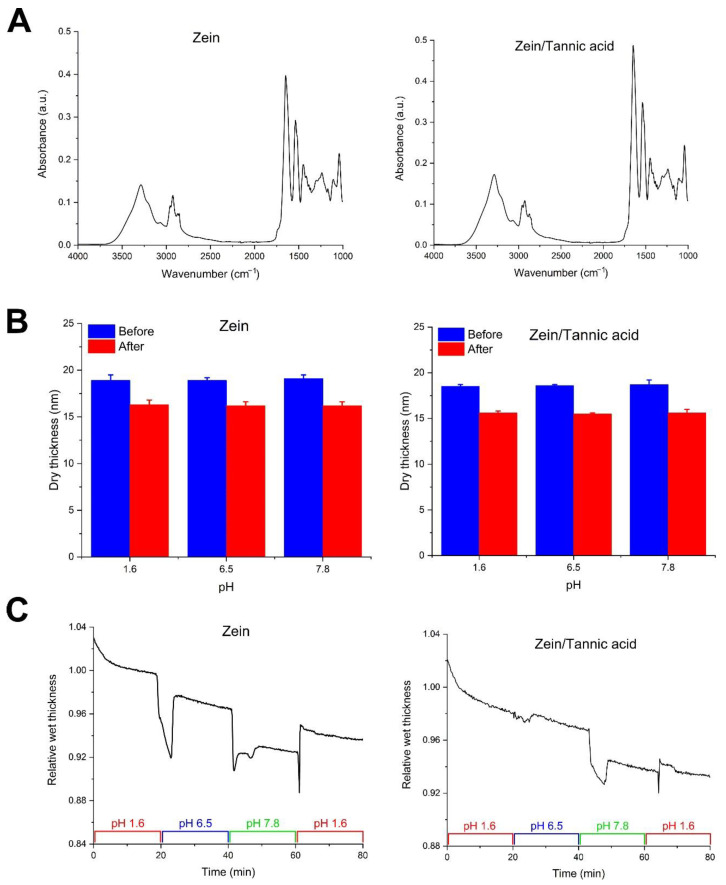
(**A**) FTIR-ATR spectra of zein and zein/tannic acid coatings. (**B**) Stability test studied using ellipsometry, where the dry thickness of the coatings was measured before and after 1 h immersion in solutions with specific pH values. (**C**) The relative wet thickness (swelling ratio, i.e., wet thickness/initial dry thickness) was measured in situ as a function of pH value over time. Data are presented as mean ± standard deviation (SD) and *n* = 3.

Figure 2A represents FTIR-ATR data for zein and zein/tannic acid coatings. Comparing zein films with and without tannic acid, no significant difference was found in the absorbance at the typical bands corresponding to the zein structure, i.e., amide A at 3288 cm^−^^1^, amide I at 1647 cm^−^^1^, amide II at 1538 cm^−^^1^, and amide III around 1242 cm^−^^1^. This observation suggests that the covalent crosslinking of the protein in the presence of low amounts of tannic acid is insignificant, which is in accordance with previous reports [37].

The pH stability and swelling properties of zein coatings, with and without tannic acid, were investigated using spectroscopic ellipsometry. As shown in Figure 2B, both the zein and zein/tannic acid coatings demonstrate a relatively small thickness reduction, i.e., dissolved matter (~15%) at all pH values. Considering the water-insoluble nature of zein, the observed mass loss may be attributed to the water-soluble impurities in the commercial zein protein [22]. We can see that the difference between the mass losses at various pH values is insignificant, suggesting that zein has a relatively weak pH responsiveness, corresponding to the mainly non-polar amino acid composition of the protein.

To examine the swelling behavior of the coatings, the wet thickness of zein and zein/tannic acid coatings was determined in situ over time as a function of pH. As shown in Figure 2C, the zein and zein/tannic acid coatings exposed to the acidic pH demonstrated a negligible swelling at first (i.e., the relative wet thickness was slightly larger than 1), yet the relative wet thickness gradually decreased, which agrees with the minor mass loss observed in Figure 2B. Similarly, other than a gradual mass loss from the coating, no swelling was found at pH 6.5 and 7.8. These observations suggest that unlike most proteins, which show a several-fold increase in thickness, i.e., swelling [38], when hydrated, the water-insoluble zein coatings possess a negligible water content regardless of pH. These results also indicate that due to its low concentration, the presence of tannic acid does not affect the pH stability and water permeability of the zein coating.

### 3.2. Optimization

**Figure 3:**
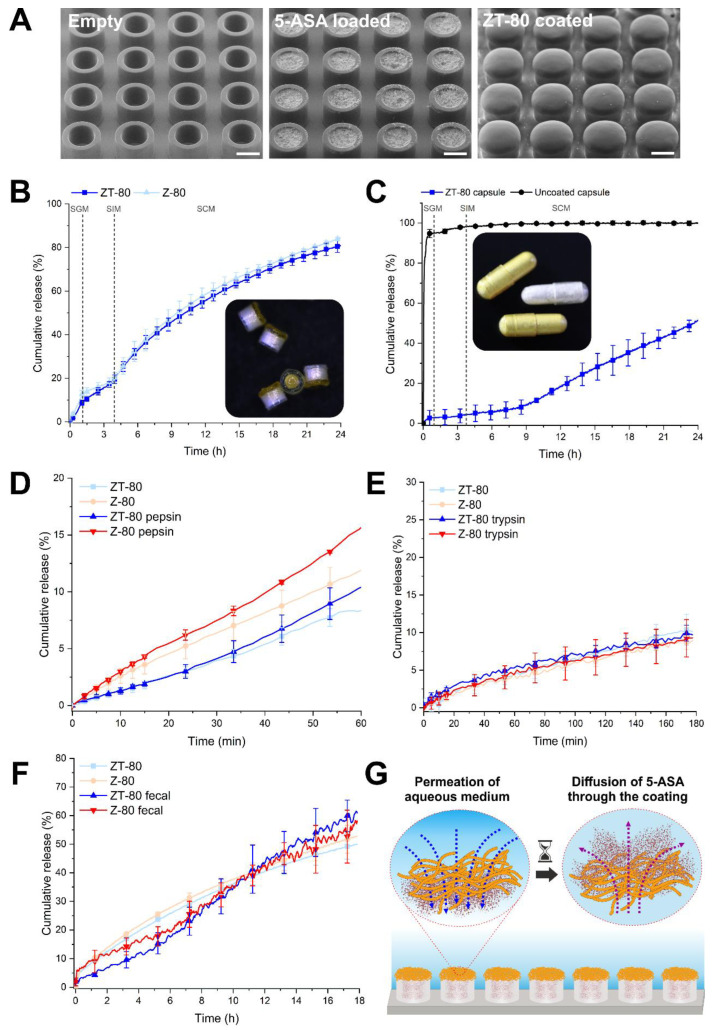
(**A**) SEM images of empty MCs loaded with 5-ASA and coated with ZT-80. (**B**) Release of 5-ASA in biorelevant media from MCs coated with ZT-80 and Z-80. Inset depicts optical microscopy images of ZT-80-coated MCs loaded with 5-ASA. (**C**) Release of 5-ASA from uncoated and ZT-80-coated capsules in biorelevant media. Inset depicts uncoated and ZT-80-coated capsules loaded with 5-ASA. (**D**–**F**) Release of 5-ASA from ZT-80 and Z-80-coated MCs in SGM holding pepsin (**D**), SIM holding trypsin (**E**), and SCM holding rat fecal contents (**F**). (**G**) Illustration of the suggested mechanism for the release of 5-ASA from ZT-80-coated MCs. Scale bars represent 200 μm. Data are presented as mean ± SD and *n* = 3.

MCs were loaded with 5-ASA (Figure 3A) and sealed with various thicknesses of zein coating, to optimize the drug release profile (Figure S1). It was observed that changing the thickness of the coating allowed tunability in the 5-ASA release profiles. In this regard, an 80 µm thick zein coating (Z-80) could provide a sustained colonic release of 5-ASA in simulated gastric, small intestinal, and colonic media (SGM, SIM, and SCM, respectively). The effect of coating thickness on the release of 5-ASA from MCs is elaborated in the Supplementary Materials, Figure S1.

The zein coatings were then functionalized with tannic acid (ZT-80, Figure 3A) and the release of 5-ASA from the coated MCs was compared with those without functionalization (Figure 3B). We found no significant difference in the release of 5-ASA from zein coatings with and without tannic acid, suggesting that tannic acid (at this concentration) has an insignificant effect on either water permeation through the coating, or the 5-ASA diffusion and rate of release from the MCs. This observation is in agreement with the results in Figure 2B,C, as well as previous reports on tannic-acid-functionalized zein [37].

In order to test the scalability of the developed coating as well as compatibility with other dosage forms, ZT-80 was applied to rat-size gelatin capsules (Figure 3C inset). Figure 3C shows the release of 5-ASA from ZT-80-coated capsules in biorelevant media in comparison with uncoated capsules, which reveals that up to 4.5 ± 3.8% of the drug was released in the gastric and small intestinal phases, followed by a slow and sustained release profile in the colonic medium. These results confirm that the developed coating can obtain the same functionality (sustained colonic release) on conventional dosage forms, thus, providing a great scalability potential.

Despite the general claim that zein protein is resistant to GIT digestive enzymes, there are many contradictory reports on the stability of the zein-based formulation against proteases [39]. Therefore, we investigated the release of 5-ASA from Z-80- and ZT-80-coated MCs in the presence of the proteolytic enzymes pepsin and trypsin (corresponding to the gastric and small intestine, respectively), as well as media containing fecal contents isolated from rats (simulating the presence of colonic bacterial enzymes). While Figure 3E,F show that the presence of trypsin and rat fecal contents has no significant effect on the release of 5-ASA from Z-80 and ZT-80, Figure 3D reveals that the tannic acid functionalization in ZT-80 can significantly decrease the rate of drug release in the presence of pepsin compared with Z-80 (*p* = 0.024). As previously reported [40], these findings suggest that tannic acid functionalization of the zein coating can improve its stability towards enzymatic digestion.

The observed lack of degradation and dissolution of the coating suggests that the developed coating performs as a hydrophobic membrane. The latter would allow slow permeation of the aqueous media into the MCs, and diffusion of the loaded drug outward thereafter, thus providing a prolonged time-dependent release profile (Figure 3G), as has been previously suggested for zein-based drug delivery systems [23]. These observations confirm the tunability, scalability, and stability of the zein/tannic acid coating and reveal its potential for the prolonged colonic delivery of 5-ASA. Thus, the effect of tannic acid functionalization on the mucoadhesion of the coating, as well as on the intestinal retention of the drug carrier, was investigated further.

### 3.3. Mucoadhesion

**Figure 4:**
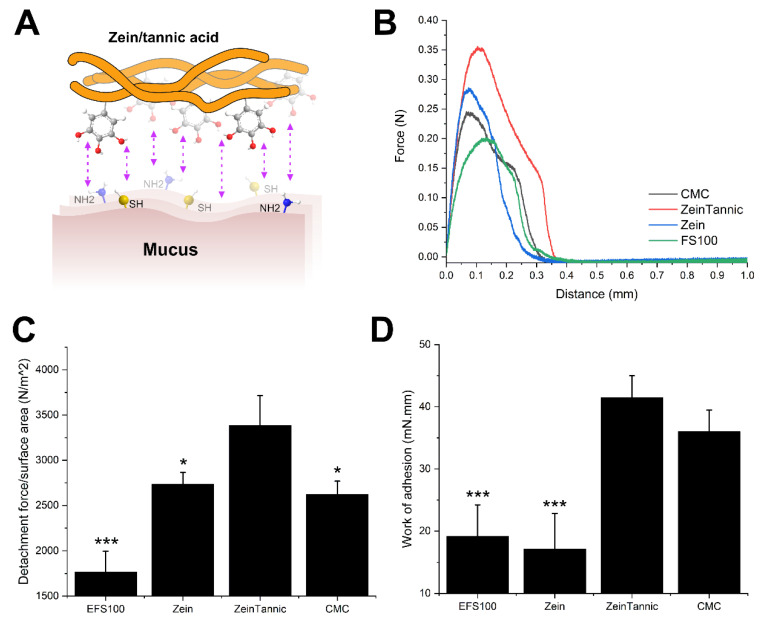
(**A**) Schematic representation of tannic-acid-mediated mucoadhesion of zein/tannic acid. (**B**) Representative force–displacement curves for the detachment of CMC, zein/tannic acid, zein and EFS100 from porcine intestinal mucus. (**C**) Detachment force of coated substrates obtained from force–displacement curves. (**D**) Work of adhesion of coated substrates calculated from force–displacement curves. Data are presented as mean ±SD, *n* = 4. Statistical significance is presented as * (*p* ≤ 0.05) and *** (*p* ≤ 0.001).

To increase the mucoadhesion of the zein coating, we hypothesized that tannic acid functionalization could facilitate the covalent bonding of the polymer to the mucus layer (Figure 4A); thus, the detachment force of the coating from intestinal mucosa was investigated. Figure 4B depicts the force–displacement curves obtained for zein/tannic acid, zein, carboxymethylcellulose (CMC, positive control [41]), and Eudragit FS100 (EFS100, commercial coating for sustained colonic delivery, negative control [42]) coatings against porcine intestinal mucosa. Figure 4C,D show the detachment force (DF) of each polymer from the mucus layer as well as the work of adhesion (WoA) derived from the force–displacement curves. Results clearly indicate that the adhesion of the zein/tannic acid to the intestinal mucus is significantly higher than EFS100 (216.6% for WoA and 191.6% for DF). Moreover, tannic acid functionalization can significantly enhance mucoadhesion compared with zein protein, and CMC as a known mucoadhesive biopolymer.

These observations indicate that the mussel-inspired adhesion mechanism can effectively take place under ex vivo conditions, which is in accordance with previous reports [43,44]. However, these experimental conditions are set in place to omit factors such as luminal flow, the presence of intestinal contents, and the clearance rate of mucus, which can influence the performance of a mucoadhesive drug carrier in vivo. Therefore, to investigate whether the same adhesion mechanism could occur under in vivo conditions, the intestinal retention of coated MCs was examined in a rat model.

### 3.4. Intestinal Retention

**Figure 5:**
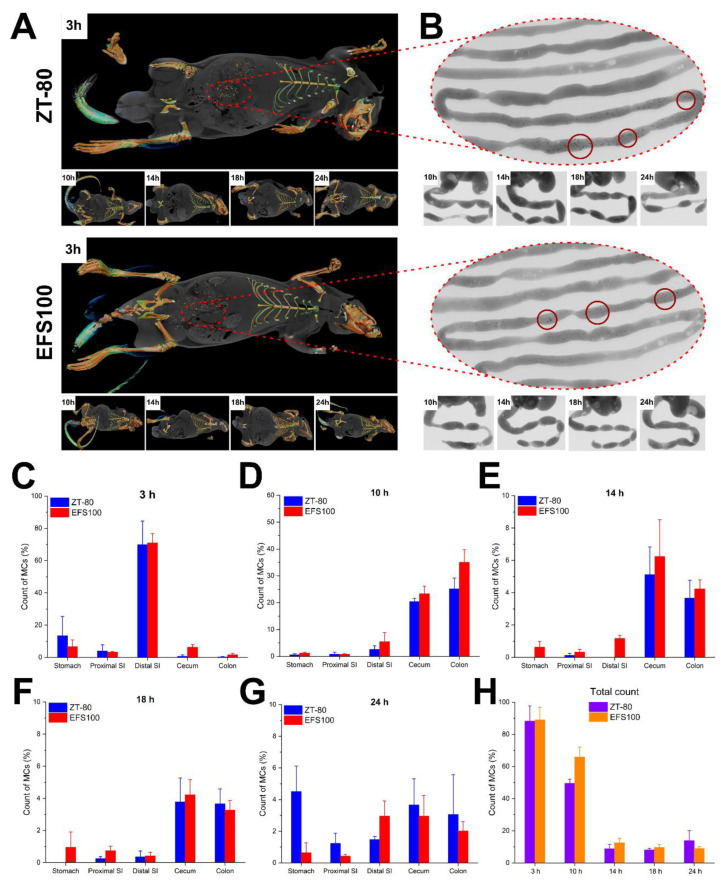
(**A**) Representative CT scan images of rats with ZT-80- and EFS100-coated MCs loaded with BaSO4. (**B**) Representative planar X-ray images of the intestines of rats. Red circles emphasize the areas with clusters of MCs. (**C**–**G**) Count of visualized MCs coated with either ZT-80 or EFS100 at different time points and the various sections of the rat GIT. (**H**) The total count of the MCs visualized inside the body of rats at different time points. Data are presented as mean ± SD, *n* = 3.

To directly correlate the ex vivo and in vivo data and fairly assess the mucoadhesive aspect of the drug carrier, an experiment was designed where the polymeric lid coating was the only variable. For this, MCs were loaded with the contrast agent, BaSO4, then coated with either ZT-80 (the mucoadhesive coating) or EFS100 (the non-mucoadhesive control), loaded into gelatin capsules, and dosed to rats. The movement of the coated MCs after the dosage was tracked and visualized inside the GIT of rats, using a previously reported method [45]. Figure 5A shows representative computed tomography (CT) scan images, while in Figure 5B we can see the corresponding planar X-ray images of the intestines of rats after dosage with the coated MCs. It is evident that the MCs are identifiable, and thus quantifiable inside the body of the rats. Moreover, based on the release profiles of MCs coated with ZT-80 and EFS100 (Figure 3B and Figure S2), we can assume that the contents remained inside the MCs during the time of this experiment.

Figure 5C–H present the count of MCs coated with either ZT-80 or EFS100 at different time points in the various sections of the GIT. It is clear that the MCs reached the distal small intestine 3 h after dosage (Figure 5C) and reached the cecum and colon after 10 h (Figure 5D). It is also evident in Figure 5E–G that less than 20% of the dosed MCs had remained inside the rat GIT after 14 h. Figure 5H shows the total count of the coated MCs inside the body of rats at different time points, which gives an overall view of their movement inside the GIT over time. Based on these results, we found no significant difference between the number of MCs coated with ZT-80 and EFS100 at various time points in the GIT sections. Therefore, these observations indicate that there was no difference between the intestinal retention time of the MCs coated with the mucoadhesive zein/tannic acid coating and that of the non-mucoadhesive EFS100 coating, despite the earlier ex vivo findings (Figure 4C,D).

These results can be attributed to the presence and flow of luminal content as well as the rapid transition and clearance of the non-adherent mucus layer, both of which can prohibit direct contact of the coated MCs with the mucus layer adherent to the epithelium [46,47]. Similar trends have previously been reported, where tannic-acid-induced mucoadhesion proved effective under ex vivo conditions but could not perform similarly in the stomach of mice due to lack of intermolecular contact with the mucus layer [48]. Based on these results, we suggest that chemically induced mucoadhesion does not have a significant effect on intestinal retention of drug carriers of sub-millimeter size (200–500 μm), and thus, the retention of the loaded active compound. However, the question remains whether our developed drug delivery system can provide sustained colonic delivery of 5-ASA. To investigate this, in vivo drug release and absorption were studied using a rat model.

### 3.5. Sustained Colon-Targeted Delivery

**Figure 6:**
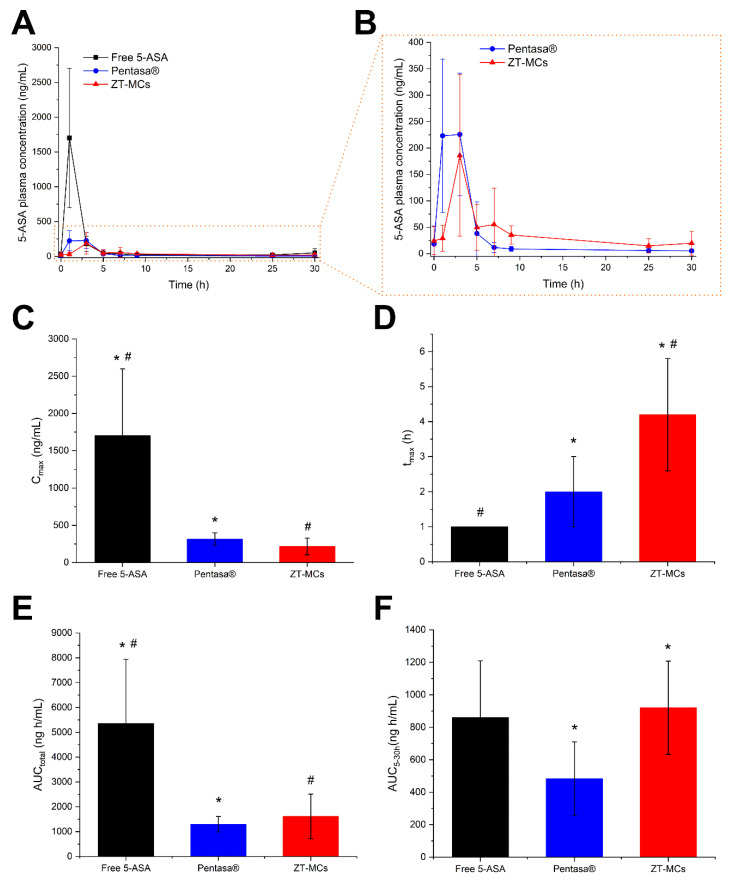
(**A**) 5-ASA plasma concentration over 30 h in rats dosed with either free 5-ASA, Pentasa^®^, or ZT-MCs filled in gelatin capsules. (**B**) Zoom in on the Pentasa^®^ and ZT-MCs profiles. Non-compartmental pharmacokinetic parameters of 5-ASA, following oral administration to rats, including (**C**) Cmax, (**D**) tmax, (**E**) AUCtotal, and (**F**) AUC5-30 h. Data are presented as mean ± SE, *n* = 5,6. *, # represent a statistically significant difference (*p* ≤ 0.05).

5-ASA was loaded into MCs, coated with the ZT-80 coating, and loaded in rat-size gelatin capsules (ZT-MCs). Free 5-ASA powder loaded into gelatin capsules was used as a negative control, while Pentasa^®^ granules [49,50] (a commercial dosage form for prolonged time-dependent release of 5-ASA), filled into gelatin capsules, were used as a positive control to compare the here-developed delivery system with a state-of-the-art formulation. Figure 6A shows the drug absorption profiles, which reveal a fast absorption and clearance of 5-ASA from the free powder samples, suggesting that 5-ASA was rapidly absorbed from the beginning of the small intestine, as expected [51]. On the other hand, Pentasa^®^ showed a prolonged absorption of 5-ASA, starting from 1 h (more clearly visible in Figure 6B), as foreseen based on previous reports on Pentasa^®^ [3,52] and the measured in vitro release profile (Supplementary Materials, Figure S3). In contrast to both controls, ZT-MCs showed a delayed and prolonged release, suggesting that the carrier had released the drug in the lower GIT.

The pharmacokinetic analysis of the 5-ASA absorption profiles shows a significant difference between the maximum plasma concentration (C_max_) of both Pentasa^®^ and ZT-MCs compared with the free 5-ASA (Figure 6C). It is important to remember that since the anti-inflammatory drug would ideally be delivered locally (in contrast to systemic delivery) to the site of inflammation in the lower GIT, a high and rapid absorption in the upper GIT is undesirable [4,6,51]. This is why the commercial formulation, Pentasa^®^, has a significantly lower C_max_ compared with free 5-ASA, which is comparable (with no significant difference) with ZT-MCs. On the other hand, we could find no significant difference between the time corresponding to the maximum plasma concentration (t_max_) of the free 5-ASA and Pentasa^®^, suggesting that both controls had started to release the drug from the beginning of the small intestine (Figure 6D). In contrast, the ZT-MCs have a t_max_ significantly higher than both controls (4.2 ± 1.6 h), suggesting a delayed release of 5-ASA. Moreover, based on the location of MCs presented in Figure 5C (3 h after dosage), we can safely assume that the 5-ASA was released from the ZT-MCs in the lower GIT (i.e., cecum and colon). Furthermore, the total area under the curve (AUC_total_) for Pentasa^®^ and ZT-MCs was significantly lower than free 5-ASA (Figure 6E), suggesting that the ZT-MCs had locally delivered a similar amount of drug to that of the commercial formulation. On the other hand, the area under the curve between 5 and 30 h (AUC5-30h) of ZT-MCs was significantly higher than Pentasa^®^ (Figure 6F), which indicates that our developed delivery system can provide a more prolonged release of 5-ASA in vivo than the commercial formulation.

It is noteworthy that despite the insignificant effect of tannic acid functionalization on the intestinal retention of MCs, the developed delivery system succeeded in providing the desired in vivo release profile for 5-ASA. Further, based on previous reports on in vivo drug absorption from microcontainers sealed with different polymeric coatings [53], we can confidently assume that the prolonged colonic delivery of 5-ASA observed here is directly the result of the release mechanism provided by the zein/tannic acid coating.

## 4. Conclusions

The anti-inflammatory drug 5-ASA was loaded into MCs and sealed with a tannic-acid-functionalized zein coating to provide a prolonged colonic release profile. The results of this study indicate:

(i) The zein/tannic acid coating can render a time-dependent prolonged release of a small molecule drug such as 5-ASA due to the hydrophobic nature of zein. in vitro results show that the developed coating can facilitate a highly tunable release profile for 5-ASA.

(ii) Functionalization with tannic acid can significantly increase the mucoadhesion of zein, which provides a chemical bonding with the mucin glycoproteins.

(iii) The chemically assisted mucoadhesion could not increase the intestinal retention of zein/tannic-acid-coated microcontainers, which is supposedly caused by the rapid clearance of mucus and luminal contents.

(iv) The zein/tannic acid coating on 5-ASA-loaded MCs provided a prolonged colon-targeted delivery of the drug in vivo, with a performance significantly exceeding that of the commercially available formulation, Pentasa^®^.

For future studies, we suggest that the here-developed 5-ASA delivery system can be investigated for the effective treatment of IBD models, e.g., ulcerative colitis. Moreover, we also suggest that the zein/tannic acid coating can be studied for application on other drug carriers such as granules and tablets.

## Data Availability

Data supporting reported results in this study can be obtained upon request to the corresponding author.

## Supplementary Materials

The supporting information can be downloaded at: https://www.mdpi.com/article/10.3390/pharmaceutics14112536/s1. Figure S1: Effect of zein coating thickness on the release profile of 5-ASA from MCs; Figure S2: Release of 5-ASA from EFS100 coated MCs; Figure S3: Release of 5-ASA from Pentasa^®^; Section S4: Fasted state simulated biorelevant media for in vitro drug release.
