# Supplementary material for: Bioadhesive Tannic-Acid-Functionalized Zein Coating Achieves Engineered Colonic Delivery of IBD Therapeutics via Reservoir Microdevices
Source: Pharmaceutics. 2022 Nov 21;14(11):2536. doi: 10.3390/pharmaceutics14112536 (PMC9699562; doi:10.3390/pharmaceutics14112536)
Supplement: Supplementary file 1 [file pharmaceutics-14-02536-s001.zip › pharmaceutics-1993361-supplementary.pdf]

Supporting Information

Bioadhesive Tannic acid-functionalized Zein Coating Achieves State-of-the-Art, Colonic Delivery of IBD Therapeutics *via* Reservoir Microdevices

Khorshid Kamguyan[1], Rolf Bech Kjeldsen[1], Saeed Zajforoushan Moghaddam[2], Melanie Randahl Nielsen[1], Esben Thormann[2], Kinga Zór[1], Line Hagner Nielsen[1], Anja Boisen[1,]*

[1]Department of Health Technology, Technical University of Denmark, Ørsteds Pl., 2800 Kongens Lyngby, Denmark

[2]Department of Chemistry, Technical University of Denmark, Kemitorvet, 2800 Kongens Lyngby, Denmark

S1. Effect of zein coating thickness on the release profile of 5-ASA from MCs

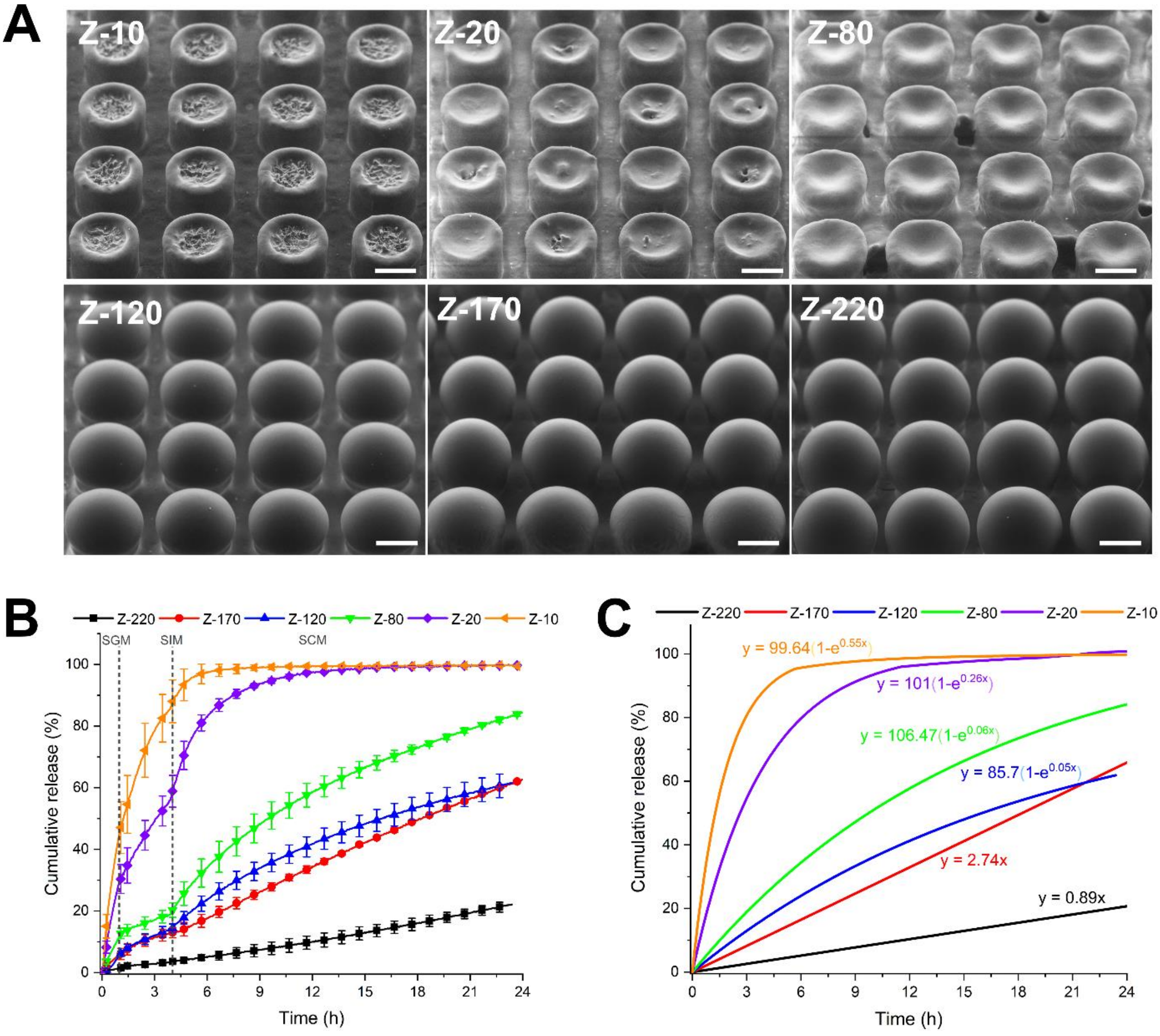

Figure S1- (A) SEM images of 5-ASA loaded MCs coated with Z-10, Z-20, Z-80, Z-120, Z-170 and Z-220 coatings. (B) Release of 5-ASA from MCs with different thicknesses in biorelevant media. (C) curve-fitting on the release profiles depicted in B. Scale bars represent 200 µm. Data is presented as mean ± SD and n=3.

Figure S1-A shows scanning electron microscopy (SEM) images of MCs loaded with 5-ASA and coated with different thicknesses of zein (10, 20, 80, 120, 170 and 220 µm). It is evident that 5-ASA is precisely packed into the MCs. However, it can be seen in the SEM images that Z-10 and Z-20 have not completely covered the loaded 5-ASA, leaving holes and imperfections in the coating. This is in contrast to Z-80 and thicker coatings, which have fully sealed the loaded drug.

Release of 5-ASA from the coated MCs was investigated in biorelevant media to study the effect of coating thickness on the drug release profiles (**Figure S1-B**). It is clear that by increasing the thickness of the coating, the rate of drug release significantly decreases from

97.42±2.63 from Z-10 to 5.00±1.40 released from Z-220 after 6 h. We can also observe in Figure 3-C that the rate of drug release is faster in gastric (pH=1.6) and colonic (pH=7.8) phases compared to the small intestinal phase (pH=6.5), which is in accordance with the swelling behavior observed in Figure 2. To better understand the differences in zein coatings with various thicknesses, a curve fitting was conducted on the release profiles (**Figure S1-C**). We can see that by increasing the thickness of the coating, the release kinetics change from a first-order release (for Z-10, Z-20, Z-80 and Z-120) to a zero-order release (for Z-170 and Z-220). The observed release kinetics suggest that 5-ASA is released through slow permeation of water through the zein membrane, dissolution of the drug inside the carrier and diffusion due to osmotic pressure.

S2. Release of 5-ASA from EFS100 coated MCs

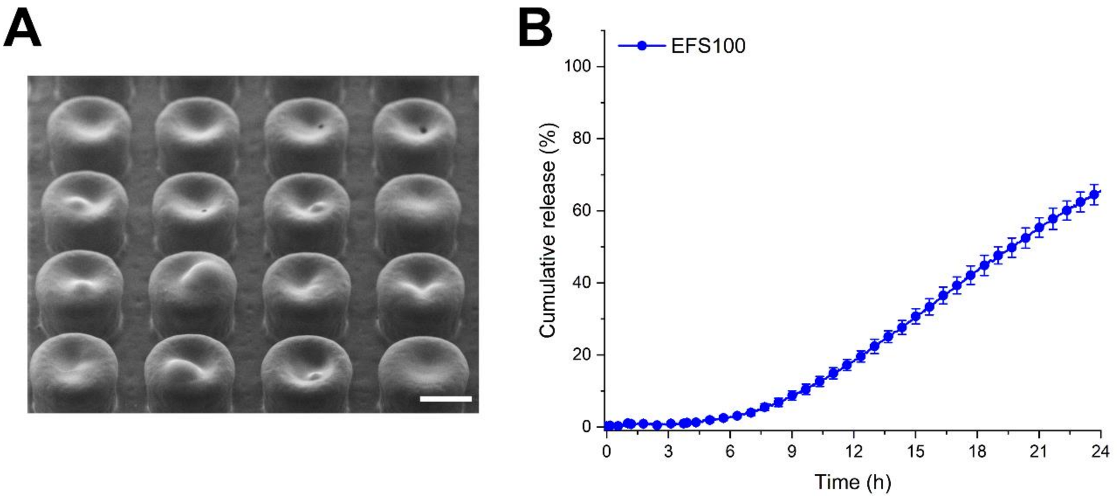

Figure S2- (A) SEM image of EFS100 coated MCs loaded with 5-ASA. (B) Release of 5-ASA from EFS100 coated MCs in simulated biorelevant media. Scale bars represent 200 µm. Data is presented as mean ± SD and n=3.

S3. Release of 5-ASA from Pentasa®

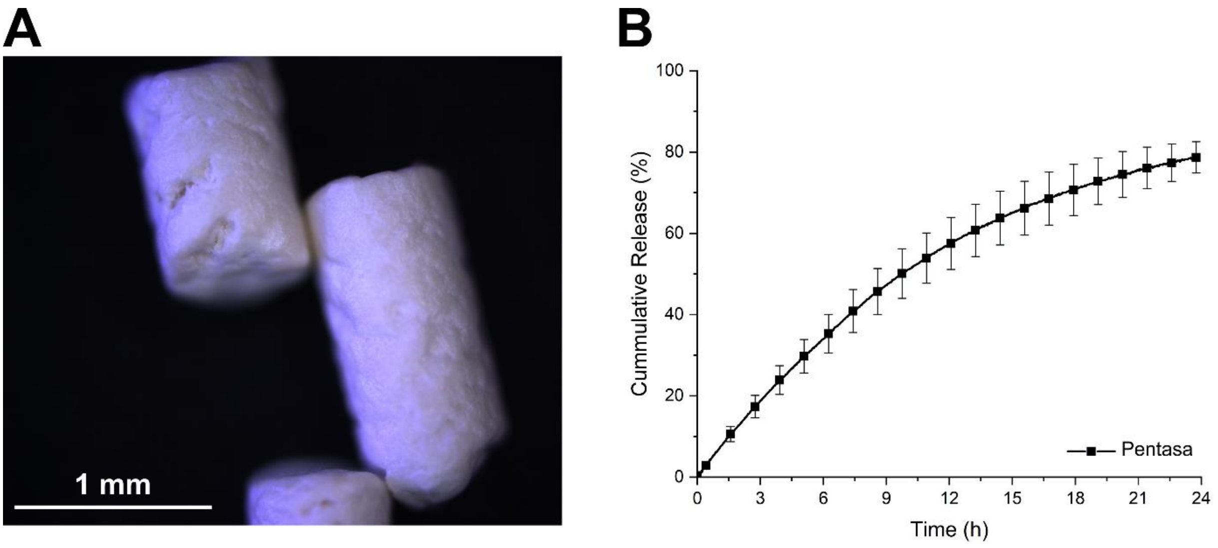

Figure S3- (A) optical microscopy image of Pentasa® granules. (B) *In vitro* release of 5-ASA from Pentasa® granules in PBS (pH = 7.4). Data is presented as mean ± SD and n=3.

S4. Fasted state simulated biorelevant media for *in vitro* drug release

SGM, pH = 1.6

**Component**	**Concentration (mM)**
**Taurocholate**	0.08
**Phospholipids**	0.02
**Sodium**	34
**Chloride**	59
**Hydrochloric acid**	25

SIM, pH = 6.5

**Component**	**Concentration (mM)**
**Taurocholate**	3

**Phospholipids**	0.75
**Sodium**	148
**Chloride**	106
**Phosphate**	29
**HEPES**	50

SCM, pH = 7.8

**Component**	**Concentration (mM)**
**Sodium cholate**	0.15
**Phospholipids**	0.3
**Oleate**	0.1
**Sodium hydroxide**	120
**TRIS**	45
**Maleate**	76
**HEPES**	50
